# Investigation of *Mycobacterium abscessus* Cluster in Hospital, Maryland, USA, 2024

**DOI:** 10.3201/eid3209.260093

**Published:** 2026-09

**Authors:** Sarah Ludmer, Monique Duwell, Tracie Harris, Surbhi Leekha, Allison Slater, Vincent Escuyer, Joseph Shea, Pascal Lapierre, Jamie Rubin, Rebecca Perlmutter, Donna Kohlershmidt, Michelle Dickinson

**Affiliations:** Centers for Disease Control and Prevention, Atlanta, Georgia, USA (S. Ludmer); Maryland Department of Health, Baltimore, Maryland, USA (S. Ludmer, M. Duwell, J. Rubin, R. Perlmutter); University of Maryland Capital Region Health, Largo, Maryland, USA (T. Harris, A. Slater); University of Maryland School of Medicine, Baltimore (S. Leekha); New York State Department of Health, Albany, New York, USA (V. Escuyer, J. Shea, P. Lapierre); Wadsworth Center, New York State Department of Health, Albany (D. Kohlershmidt, M. Dickinson)

**Keywords:** Mycobacterium abscessus, antimicrobial resistance, tuberculosis and other mycobacteria, bacteria, opportunistic infections, nontuberculous mycobacteria, hospitals, environmental exposure, biofilms, water microbiology, molecular epidemiology, whole genome sequencing, disease outbreaks, infection control, Maryland, United States

## Abstract

*Mycobacterium abscessus* infections are frequently drug resistant and can cause severe disease, particularly among vulnerable populations in healthcare settings. In June 2024, a cluster of *M. abscessus* infection cases was reported at a hospital in Maryland, USA. An investigation involving hospital infection prevention staff, the Maryland Department of Health, the Centers for Disease Control and Prevention, and the Wadsworth Laboratory of the New York State Department of Health included initiation of mitigation efforts such as tap water use restrictions, point-of-use water filters, hospitalwide water flushing, and staff education regarding sink safety. Whole-genome sequencing of patient and environmental isolates, coupled with epidemiologic data, revealed a strong association (0–24 single-nucleotide polymorphism differences) between environmental and patient samples, indicating that infections were likely a result of patient exposure to *M. abscessus*. Ensuring initiation of mitigation initiatives likely prevented additional patient infections. Although effective in this investigation, not all mitigation strategies are equal or sustainable.

Nontuberculous mycobacteria (NTM) are a group of bacteria found naturally in soil, dust, and water; infections caused by NTMs are increasing globally (*1**–**3*). The NTM *Mycobacterium abscessus* and its subspecies are classified as rapid growers and are responsible for skin and soft tissue infections, bacteremia, and pulmonary infections, among other conditions (*4*). *M. abscessus* infections are difficult to treat because of intrinsic drug resistance that limits treatment options (*2*). The environmental persistence of *M. abscessus* and the often treatment-resistant infections it can cause make it an emerging bacterium of concern. *M. abscessus* outbreaks have been reported in healthcare settings, often involving exposure to contaminated water sources (*5**–**8*). 

In June 2024, hospital infection prevention (IP) staff at a hospital in Maryland, USA, notified the Maryland Department of Health (MDH) of 10 patients with cultures positive for *M. abscessus*. The ensuing investigation involved the IP staff, MDH, the Centers for Disease Control and Prevention (CDC), and the Wadsworth Laboratory of the New York State Department of Health. This activity was reviewed by the CDC Public Health Infrastructure Center’s human subjects review staff, was deemed not research, and was conducted consistent with applicable federal law and CDC policy (e.g., 45 C.F.R. part 46, 21 C.F.R. part 56; 42 U.S.C. §241(d); 5 U.S.C. §552a; 44 U.S.C. §3501 et seq.).

## Methods

### Epidemiologic Investigation

After they identified an initial case of *M. abscessus* infection in May 2024, hospital IP staff initiated retroactive case finding. The hospital had a previous cluster of 3 cases of *M. abscessus* (2 positive CSF cultures, 1 positive sputum culture) in 2021, shortly after occupancy of a newly constructed building. Preoccupancy water quality testing was within acceptable ranges but did not include specific testing for *M. abscessus*. With guidance from MDH and CDC, water samples from the 2021 outbreak were tested at CDC, where NTM was identified, but not *M. abscessus*. 

In the cluster reported in 2024, a total of 10 patients had *M. abscessus* identified from clinical cultures during March 28, 2023–June 5, 2024. Hospital IP staff identified 1 additional patient in September 2024 and reported that case to MDH, bringing the total to 11 patients. The hospital implemented mitigation strategies after the first 2 cases were identified in 2024, including hospitalwide water flushing, increased point-of-entry supplemental disinfectant (monochloramine) levels, restrictions on use of tap water and ice machines, and staff education and monitoring of sink safety. The hospital also installed PALL point-of-use filters (https://www.pall.com) in sinks and medication rooms in high-risk patient care areas, including intensive care unit patient rooms, operating room (OR) scrub sinks, and cancer centers. The hospital water management plan guided some of the mitigation strategies and was ultimately updated to include more frequent building flushes, residual disinfectant testing, and inclusion of *M. abscessus* in water microbial testing. All those measures have been shown to reduce risk for patient exposure to contaminated water sources (*6*). Investigators also collected information regarding timing of clinical culture collection, patient location within the facility, and clinical manifestations (including patient procedures).

### Laboratory Testing

Before initiating mitigation strategies, the hospital water management team completed environmental sampling to attempt to identify potential sources of *M. abscessus* in the hospital. Water sampling included bulk water samples (1 L, mixed hot and cold) and swab specimens from faucets, sinks, and ice machines; testing was conducted by an accredited commercial environmental microbiology laboratory. The team chose locations for water samples on the basis of patient location in the hospital, high-risk patient care areas, and sources implicated in reports of other *M. abscessus* outbreaks (*5*). In addition to cultures, supplemental disinfectant levels were measured for faucets inside patient rooms, medication rooms, ice machine water, and OR scrub sinks. Heater-cooler machines have been implicated in other outbreaks of NTM (*8**–**9*), and a bulk water sample from a heater-cooler machine (CardioQuip, https://www.cardioquip.com) used during 1 patient’s hospitalization was included; that case-patient was the only one with heater-cooler machine exposure. After mitigation methods were implemented, resampling was conducted to determine effectiveness. For point-of-use filters, sampling was done before and after filter installation.

### Whole-Genome Sequencing

Whole-genome sequencing (WGS) was performed at the New York State Department of Health Wadsworth Center for both clinical and environmental isolates using the NextSeq platform (Illumina, https://www.illumina.com) (*10**, **11*)*.* De novo genome assemblies were performed using the Shovill pipeline version 1.1.0, default parameters (https://github.com/tseemann/shovill). No postmitigation isolates were sequenced.

## Results

### Epidemiologic Investigation

Among 11 patients identified, median age was 40 (range 24–76) years. Five patients underwent surgical procedures; 2 had procedures in OR A, 2 in OR B, and 1 in OR C. No patients had the same procedure; however, the 2 patients in OR A each had cardiac surgery. One patient had a heater-cooler machine used during the cardiac procedure. Two rooms housed multiple affected patients; however, their admissions did not overlap in time. Otherwise, we identified no shared patient ward or unit locations. Six (55%) of the 11 patients were treated with antimicrobial therapy as directed by infectious disease physicians, after positive culture results. One (9%) patient died within 60 days of the first positive culture; that death was not attributed to *M. abscessus* infection.

### Laboratory Testing

Among culture samples from 11 patients, 6 (55%) were from sputum samples, 4 (36%) from blood samples, and 1 (9%) from an abdominal abscess. Before initiation of mitigation strategies, the IP team collected 47 environmental samples from 19 locations throughout the hospital. Nineteen were bulk water samples, and 28 were swab specimens from faucet and sink drains, including ice machines. Testing revealed *M. abscessus* in the water system at different levels throughout the hospital building, including in ice machines. Among all samples, 18 (38%) grew *M. abscessus* and 15 (32%) had acid-fast bacilli but not *M. abscessus*. For samples that grew *M. abscessus*, density varied: 10–48,000 CFU/swab, or <1–39 CFU/mL for bulk water samples. The sample from the CardioQuip heater-cooler machine did not yield any bacteria. An investigation into various uses of tap water and ice within the hospital did not identify any obvious source of specimen contamination from tap water or ice during specimen collection or processing. However, investigation of case 11, which was identified after mitigation efforts began, revealed ice from restricted ice machines was used to store saline syringes that were used during bronchoalveolar lavage.

After review of clinical and epidemiologic data, we determined that 6 patients had clinical infections and 5 represented likely colonization or specimen contamination. All 5 of those cases involved respiratory tract cultures (Table), thought to be colonization or the result of specimen contamination either because the specimen collection was completed in the emergency department before admission and patients had no previous exposure to the facility or because they had respiratory illness at admission. 

**Table:** Summary of patient characteristics, including pertinent investigation results, from *Mycobacterium abscessus* cluster in hospital, Maryland, USA, 2024*

Patient ID	Age, y/sex	Risk factors or underlying conditions	Patient unit	Specimen type	Culture date	OR procedure	OR	Matched to ENV isolate	Type
A	30/F	Gastronomy tube	1	Blood	2023 Mar 28	Laminectomy	B	Y	Clinical case
B	38/F	No significant risk factors/conditions	1	Abdominal abscess	2023 Mar 30	Cholecystectomy	B	Y	Clinical case
C	34/F	Endocarditis, hepatitis C	2†	Sputum	2023 Aug 22	None	NA	Y	Contaminant/colonization
D	74/F	Tuberculosis, diabetes mellitus, coronary artery disease	3	Sputum	2023 Sep 9	None	NA	Y	Contaminant/colonization
E	32/F	Sickle cell disease	3, 5	Blood	2023 Sep 13	None	NA	Y	Clinical case
F	24/M	Pericarditis	2,‡ 4	Sputum	2023 Nov 27	Creation pericardial window	A	N§	Contaminant/colonization
G	76/M	Coronary artery disease	2†	Blood	2024 Feb 8	CABG	A	Y	Clinical case
H	74/F	Tracheostomy, gastronomy tube, endocarditis, lung cancer, sarcoidosis	1, 2,‡ 3	Blood	2024 Apr 2	Bilateral aortogram, iliac stents, brachial artery repair, femoral angioplasties	C, D, E	Y	Clinical case
I	42/M	No significant risk factors/conditions	6	Sputum	2024 Apr 2	None	NA	Y	Contaminant/colonization
J	72/F	Necrotizing pancreatitis, kidney and breast cancer, diabetes mellitus	4	Blood	2024 Jun 5	None	NA	NA	Clinical case
K	53/F	HIV, tuberculosis, diabetes mellitus	3	Sputum	2024 Sep 18	None	NA	N§	Contaminant/colonization

*Unit names are deidentified; placeholder numbers have been assigned to maintain anonymity. ENV, environmental; NA, not applicable; OR, operating room. †Patients shared the same room on unit 2 but did not overlap in admission dates. ‡Patients shared the same room on unit 2 but did not overlap in admission dates. §Only matched to other patient isolates.

The hospital installed point-of-use filters in several locations, guided by the epidemiologic investigation, including high-risk patient areas, ORs, and intensive care units. Water sampling after installation of point-of-use filters showed no bacterial growth. Repeat sampling of sink and faucet sites after flushing and increasing point of entry supplemental disinfectant revealed optimal residual disinfectant but continued detection of *M. abscessus* to different degrees.

### WGS

All 18 environmental samples that grew *M. abscessus* and 10 available clinical isolates from 10 distinct patients were sent to the Wadsworth Laboratory for WGS. Sequences reported in this article were submitted to the National Center for Biotechnology Information BioProject Database (BioProject accession no. PRJNA1403517). Among the 28 samples sequenced, we identified 5 subclusters, consisting of 10 patient isolates and 17 environmental isolates. Maximum whole-genome single-nucleotide polymorphism (SNP) distance of the core genome across the 28 isolates was 98,252. Within each subcluster, associated subcluster isolates were within 0–24 SNPs, indicating a high degree of relatedness (*12**–**13*). Four subclusters each included both patient isolates and environmental isolates (0–15 SNPs), and 1 included only isolates from patients (20–24 SNPs). One patient isolate identically matched an environmental sample from the postanesthesia care unit (PACU) ice machine (0 SNP difference). The patient had been in the PACU after a procedure for <24 hours. Two other patient isolates showed close association with PACU environmental samples. One showed close association (5 SNPs) to a PACU sink water sample; that patient had 3 operations during hospital admission. The other patient, whose isolate was closely associated (11 SNPs) with a PACU sink faucet swab, had not spent any time in the PACU and was ultimately determined to be a likely specimen contamination because the sample was collected in the emergency department before admission. Two patient isolates were not found to be closely associated with any of the environmental samples but did cluster closely (20–24 SNPs) with a patient isolate from the facility in 2021. However, we could not determine any additional epidemiologic link between the 2 isolates from this cluster and the 2021 isolate. One environmental isolate was not closely related (>80 SNPs) to any other environmental or patient isolates.

## Discussion

The infectious source in hospital outbreaks of NTM can be hard to identify (*5*). Water sampling and subsequent sequencing of clinical and environmental isolates was instrumental to confirm colonization of the water system in this hospital and implicate ice machines as the probable source of infection on the basis of the close associations (low SNP differences) observed between clinical and environmental samples. The use of genetic sequencing and epidemiologic investigation in outbreaks like this one enables investigators to identify current and potential future sources of pathogen exposure, target interventions, and efficiently allocate resources. Ice machines have been identified as a potential source of NTM infection in other outbreaks, and investigators should consider possible contamination or colonization in future outbreaks (*7*,*14*). 

*M. abscessus* is challenging to remove from a water system after a biofilm has developed (*9*). After *M. abscessus* is identified within a water system, immediate mitigation measures are needed because the persistent organisms can cause serious and difficult to treat infections. Swift action by hospital staff to remove potential sources of infection from patient areas was likely key to preventing additional infections in this cluster. Point-of-use filters are effective; however, they are expensive and must be inspected and replaced regularly. Building flushing and increasing supplemental water disinfectant levels showed a temporary decrease in *M. abscessus* levels. Continued detection could indicate biofilm formation or organism resistance to disinfection practices or agents (e.g., monochloramine), making water system eradication a challenge. 

Infections with *M. abscessus* at this facility first occurred within a few months of initial occupancy of a new building, designed to be Leadership in Energy and Environmental Design–certified. New hospital buildings, particularly those with lower water flow and recirculation inherent to energy-efficient design, have been associated with NTM outbreaks (*6*,*15*). Adherence to a robust water management plan and sink safety education are vital practices to prevent outbreaks from *M. abscessus* and other opportunistic premise-plumbing pathogens.
